# From discomfort to danger: Exploring how affective obstacle properties influence avoidance in stepping

**DOI:** 10.1177/03010066251360582

**Published:** 2025-07-30

**Authors:** Zhong Jian Chee, Martin Giesel, Constanze Hesse

**Affiliations:** 1019 University of Aberdeen, UK

**Keywords:** obstacle avoidance, material perception, affective properties, surface properties

## Abstract

Stepping over obstacles requires adjusting the foot trajectory to avoid contact with surfaces that may be hazardous or unpleasant to step on. While it is well established that obstacle height and stability influence stepping behaviour, little is known about how perceptual affective evaluations, such as dangerousness, unpleasantness, and painfulness, modulate avoidance strategies. In Experiment 1 (*N* = 20), participants stepped over obstacles covered with stones varying in size and density while rating their perceived unpleasantness. Visual uncertainty was manipulated by comparing monocular and binocular viewing. Lead minimum foot clearance (MFC) was initially higher under monocular vision but decreased to binocular levels over trials. While obstacle unpleasantness did not systematically affect MFC or crossing step length, perceived unpleasantness ratings correlated weakly with crossing step length. However, because dangerousness and painfulness ratings were not collected, it remained unclear whether unpleasantness directly influenced avoidance behaviour or served as a proxy for perceived risk. To address this, Experiment 2 (*N* = 22) introduced obstacles covered with metal stud spikes or smooth surfaces, with additional ratings of dangerousness and painfulness. Results showed that MFC was higher for spiky than smooth obstacles. Crucially, in this experiment, ratings of perceived dangerousness, not unpleasantness, correlated positively with crossing step length, after controlling for other perceptual ratings. These findings suggest that perceptual affective properties modulate avoidance parameters. However, the nature of those modulations is stimulus specific and highly depends on task demands.

## Introduction

Imagine having to step over an obstacle like a branch of brambles or some dandelions that have grown over the footpath. Would you adjust the way you step over them, depending on how unpleasant you judge potential contact with the obstacles to be? If and how we adjust our movements in response to the perceived unpleasantness of an obstacle was the primary question we aimed to explore in this study.

Nonpainful unpleasantness is a negative affective sensation that, as implied by the name, feels unpleasant without eliciting pain. Regarding the question of which physical material properties tend to be perceived as unpleasant, previous studies have consistently found that there is a strong association between roughness and the perceived unpleasantness in both the visual (e.g., Van Egmond et al., 2009) and tactile domains (e.g., Drewing et al., 2018; Ekman et al., 1965; Kitada et al., 2012; Lin et al., 2023; Verrillo et al., 1999). This positive correlation between roughness and perceived unpleasantness has been found using natural (e.g., sandpaper and fabrics) and artificial (i.e., raised 2-D dot patterns) surface variations. Generally, surface roughness is a complex texture property that depends on several physical parameters such as the size, density, spacing, and jaggedness of the particles on surfaces (Lederman & Abbott, 1981; Lederman & Taylor, 1972). Roughness is primarily determined visually and/or haptically (but can also be assessed auditorily; see Guest et al., 2002), and assessments in the visual and haptic modalities tend to be highly correlated (e.g., Bergmann Tiest & Kappers, 2007; Binns, 1937; Kangur et al., 2022).

Given the well-known link between roughness and unpleasantness and the fact that surface roughness can be varied by manipulating the size and density of the elements on a surface, we created obstacles with surfaces covered in large and small stones that were either sparsely or densely spaced (Experiment 1). We assessed participants’ visual judgements of their roughness and unpleasantness on touch via a perceptual rating task. Our primary interest was to determine whether participants’ perception of roughness and/or unpleasantness of the obstacle surfaces affected their movements when stepping over them.

There are several reasons to expect an effect of perceived unpleasantness on our actions. Visuomotor control can be described as a decision-making process under uncertainty (e.g., Trommershäuser et al., 2003; Wolpert & Landy, 2012). To make safe movements, we must consider uncertainties during the action planning process. In this context, there are two main sources of uncertainty: perceptual uncertainty and motor uncertainty. For example, in an obstacle avoidance task, the visual information about the position of a potential obstacle or its dimensions might be unreliable because of low light levels, resulting in an increase in perceptual uncertainty. However, even if the visual information is reliable enough to allow us to plan a perfectly safe action, we still might not be able to flawlessly execute this action because we have limited control over the precision of our movements due to motor noise (i.e., motor uncertainty). The precision of our actions might be further reduced by time constraints that require us to move quickly. The degree to which we must consider uncertainties during movement planning and execution is modulated by the costs or consequences that could result from potential mistakes during action execution, such as stepping on, or colliding with, a fragile or dangerous obstacle. Potential costs will have to be weighed against the potential gains of moving quicker or more efficiently.

The latter issue of minimising the negative consequences of potential action mistakes has been studied in obstacle avoidance for both hand (i.e., reaching and grasping) and leg (i.e., stepping and walking) movements (de Haan et al., 2014; Giesel et al., 2020; Kangur et al., 2017; Patla et al., 1996). Generally, it has been found that as the potential costs of collisions increase (such as potential spillage or breakage), humans tend to perform more pronounced avoidance responses – primarily reflected in keeping larger distances between the moving limbs and the obstacles, i.e., safety margin (e.g., de Haan et al., 2014; Giesel et al., 2020; Patla et al., 1996). Furthermore, participants may overall move slower to ensure more accurate movements if the cost of collisions is high (e.g., Jansen et al., 2011; Kangur et al., 2017). With respect to perceived unpleasantness, it stands to reason that potential contact with a more unpleasant surface is associated with a higher cost (i.e., negative consequence) and hence that avoidance responses may be more pronounced.

A further reason for assuming that perceived unpleasantness may affect avoidance responses comes from studies that have observed effects of affective responses on the perception of metric properties such as distance and size of objects. For example, threatening stimuli have been found to be perceived as being physically closer than nonthreatening stimuli (Cole et al., 2013). Similarly, in a recent study by Sun et al. (2021), a *neutral* target object was perceived as being closer and larger when it was placed on top of a toy rat (negative valence) than when the same target was placed on top of a toy squirrel (positive valence) or a wooden block (neutral valence). However, if and how those changes in visual perception affect associated actions to those objects remains unclear (see Sun et al., 2021). So far, the question of how pleasant and unpleasant stimuli affect our behavioural responses has largely been investigated using image-based reaction-time tasks (i.e., approach-avoidance paradigms). Using those paradigms, it has commonly been found that participants are faster to avoid unpleasant/aversive stimuli (e.g., spiders; Rinck & Becker, 2007) and approach pleasant/appetitive stimuli (e.g., food; Kahveci et al., 2020). Regarding the effects of affective responses induced via images on stepping and gait movements, studies generally observed an initial “freezing” response (e.g., reduced body sway) towards unpleasant images and a facilitation of gait initiation (e.g., increased step velocity, displacement, and velocity of centre of pressure) towards pleasant images (e.g., Azevedo et al., 2005; Naugle et al., 2010, 2011; Stins & Beek, 2011; Yiou et al., 2014), again mirroring approach-avoidance tendencies for pleasant and unpleasant stimuli, respectively. More pertinent to obstacle avoidance, Caffier et al. (2017) projected threatening and nonthreatening image slides on the left side of the participants, whilst they initiated a short walking movement. They found that participants showed an avoidance-like response by veering away from the screen when threat-eliciting images were displayed.

However, most studies looking at the link between affective responses and actions primarily employed images that were not directly task relevant. Here, we are specifically interested in determining whether and how stepping parameters are affected when participants step over obstacles varying in perceived unpleasantness (on contact). Drawing from the findings using images, we expected larger avoidance responses for obstacles that are perceived as more unpleasant. To determine the size of the avoidance response, we focussed on two main stepping parameters: minimum foot clearance (MFC) and crossing step length (Buckley et al., 2010; Loverro et al., 2013). We hypothesised that the MFC and crossing step length increase for obstacles perceived as being more unpleasant. Analysis were conducted separately for the leading leg (i.e., lead MFC; the leg crossing the obstacle first under visual guidance) and the trailing leg (i.e., trail MFC; the leg crossing the obstacle once the leading leg has cleared the obstacle), as previous studies demonstrate that primarily the leading leg is affected by the potential costs of collision (Patla et al., 1996) and the availability of visual information (Mohagheghi et al., 2004).

Furthermore, as discussed above, visual uncertainty is considered an important factor that critically determines the execution and accuracy of our actions (Jarbo et al., 2018; Landy et al., 2007; Schlicht & Schrater, 2007). In obstacle avoidance, increased visual uncertainty generally results in larger safety/clearance margins in both foot (e.g., Hayhoe et al., 2009; Patla et al., 2002) and hand movements (e.g., Cohen et al., 2010; Giesel et al., 2020). Specifically, it has been argued that for accurate actions, the availability of binocular information is essential (Chapman et al., 2012; Giesel et al., 2020; Melmoth & Grant, 2006; Servos et al., 1992; Zhao & Allison, 2021). Generally, perceptual uncertainty increases under monocular viewing, and humans tend to compensate for this uncertainty by increasing safety margins. Consequently, we expected that the lead MFC (under visual control) would be higher in the monocular condition than in the binocular condition. Furthermore, there is evidence that (slant-mediated) surface roughness judgements are inferior with monocular vision (Allison et al., 2009). Given those findings, we wanted to further explore if monocular vision also impairs the estimation of surface properties, potentially resulting in attenuated effects of perceived roughness/unpleasantness on stepping kinematics under monocular viewing conditions as compared to binocular viewing. Finally, as stepping kinematics have been found to change with practice/repetition (Heijnen et al., 2012; Rhea et al., 2010), we also explored the effects of trials on MFC and crossing step length. If participants become more confident over trials, we would expect MFC to decrease. We did not have any specific predictions for the effects of trials on crossing step length.

## Experiment 1

### Method

#### Participants

Thirty-one naïve participants were recruited. Eleven participants had to be excluded due to technical issues (see the Data Analysis section for more details). Hence, the following results were based on 20 participants (12 females and 8 males). We decided a priori to stop collecting data when we had 20 valid participants, and this sample size estimate was based on previous literature on stepping over obstacles (e.g., Buckley et al., 2010; Heijnen et al., 2012; Muir et al., 2020). Their age ranged from 17 to 40 years (*M* = 21.85, *SD* = 5.27) with a mean height of 170.2 cm (*SD* = 12.08) and a mean leg length of 97.6 cm (*SD* = 5.68). All participants were able to walk continuously without difficulties and had normal or corrected-to-normal visual acuity. Twelve were left-eye dominant and eight were right-eye dominant. This study complied with the Ethics Code of the British Psychological Society (BPS) and was approved by the Psychology Ethics Committee of the University of Aberdeen (ID: 694797). Participants provided written informed consent before the experiment and were reimbursed either with course credits or £8/hour upon completion.

#### Stimuli and Apparatus

**Figure 1.**
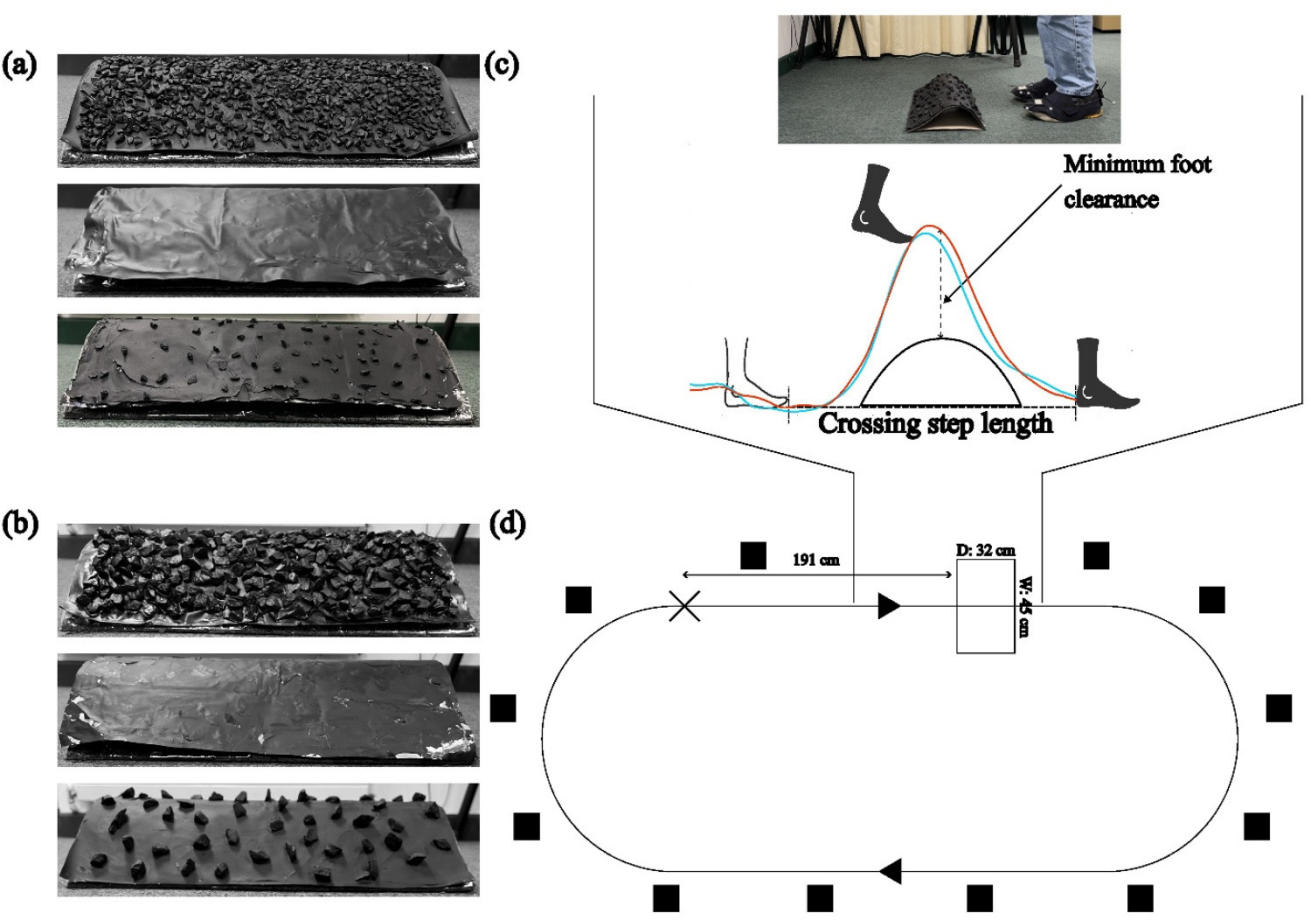
Obstacles with (a) small stones: dense, flipped, sparse, and (b) large stones: dense, flipped, and sparse. (c) An experimenter standing in front of the obstacle with markers on both feet and a representative participant’s time-normalised trajectories (average across 90 trials) of the leading foot over the obstacle under monocular (red line) and binocular conditions (cyan line) with the obstacle shown in cross-section. The shaded foot represents the leading foot, and the nonshaded foot represents the trailing foot. (d) Top view of the experimental layout. The cross represents the starting position, the open rectangle represents the obstacle, the black arrows show the direction of walking, and the black boxes represent the cameras. The figure is not drawn to scale.

The obstacles were made of surfaces covered with two differently sized stones (i.e., small or large) in two different manners (i.e., sparse or dense). In the dense condition, the whole obstacle surface was closely covered in stones (requiring about four times as many small stones as large stones to achieve 100% coverage). In the sparse condition, a smaller number of stones was evenly distributed over the whole surface (using about twice as many small stones as large stones) resulting in about 20% coverage for the large stones and 10% coverage for the small stones. The obstacles are referred to as dense-large, dense-small, sparse-large, and sparse-small hereafter. Two additional conditions where the surfaces with large or small stones were flipped upside down to present a smooth surface while maintaining the same height were included (i.e., smooth-large or smooth-small obstacles; see Figure 1a and b). The smooth surfaces were included to serve as baseline conditions to account for interindividual differences such as leg length and variations in gait patterns (see the Data Analysis section for more details). We treated each combination of stone size and density as an obstacle type, hence resulting in six unique obstacles. The width (45 cm) and depth (32 cm) were the same for all obstacles. The maximum height of the small-stone obstacles and the large-stone obstacles was ∼9.3 cm and ∼9.9 cm, respectively.

Markers were attached to the top of the foot (i.e., centrally above the metatarsals) and just above the heel (calcaneus) of participants’ feet wearing felt slippers over their shoes (see Figure 1c). In the following, we will refer to those markers as “toe” and “heel” markers. The markers were captured by a 12-camera passive infrared motion tracker (OptiTrack, Flex 3) at 100 Hz using the Motive software. The experiment was programmed in MATLAB (MathWorks, Natick, MA, USA), and data were extracted using custom-written MATLAB scripts.

#### Procedure

An eye-dominance test was conducted at the beginning of the experiment. We instructed participants to stretch their arms in front of them and create a triangular aperture between their thumbs. With both eyes open, participants were asked to position the middle of the triangular aperture such that a red dot on a wall at a distance of ∼2.75 m was in its centre. Participants then closed one eye after the other, and the eye for which the dot stayed closer to the centre of the aperture was determined as dominant. Afterwards, relevant demographic (i.e., age and gender) and anthropometric information (e.g., height and leg length) were collected.

The experiment consisted of a stepping (obstacle avoidance) task and a rating task. The stepping task always preceded the rating task. The stepping task was divided into two blocks: one completed with binocular vision and one with monocular vision. Participants wore an eye patch over their nondominant eye for the monocular condition. We counterbalanced the blocks so that half of the participants walked with binocular vision first and the other half walked with monocular vision first. Due to participant exclusions, 11 participants started with the monocular vision condition and nine with the binocular vision condition. Each block contained 90 trials, with 15 trials for each type of obstacle.

Before starting the stepping task, participants were asked to stand in a fixed position and face away from the obstacle (see Figure 1d). This was done to prevent participants from seeing the obstacle the experimenter was placing. The experimenter then asked participants to turn around and face the obstacle. Three successive beep sounds were played to signal the participants to start walking. We instructed participants to walk as naturally as possible at a self-selected pace and continuously along the circuit until 15 trials of stepping over an obstacle were completed. Participants typically took two to three steps from the starting point before stepping over the obstacle (see Figure 1d). Data was only recorded from the starting point to approximately one step after the obstacle. We did not record data when participants were making turns or when they were on the return path. A beep sound was played to signal participants to stop walking after 15 trials. Participants were instructed to stand behind the starting position again before starting to walk for the next obstacle. Due to a programming error, the random number generator started from the same seed for some participants, resulting in some obstacle presentation orders being repeated across participants. Post hoc tests with obstacle presentation order as a fixed effect in mixed models suggested that this had no effect on the MFC or crossing step length.

For the rating task, participants were instructed to sit on a chair placed at the starting position of the stepping task and visually rate the obstacles that were placed in the same position as in the stepping task (excluding the two smooth surfaces) on perceived unpleasantness, roughness, hardness, height, and density by pointing with a mouse on a continuous scale between 0 and 100. Specifically, for ratings of unpleasantness, we asked participants to imagine how unpleasant it would be to step on the surface of the obstacle. However, for the ratings of roughness, hardness, height, and density, we asked participants to consider the elements (i.e., stones) on the obstacle. The presentation of obstacles for rating was randomised, and each obstacle was rated only once. There were no practice trials for both stepping and ratings, and the entire experimental session lasted for approximately 90 min.

#### Data Analysis

Kinematic data were filtered with a second-order Butterworth filter with a cut-off frequency of 15 Hz. We extracted kinematic data around the area of interest, which was the region roughly one step before and after the obstacle. Eleven participants were excluded from all analyses due to substantial missing data around the area of interest, which made it impossible to compute our variables of interest. For the remaining 20 participants, 60 trials (1.67%) out of 3600 trials were missing due to obstruction of markers during data collection and hence excluded. We computed the MFC by taking the position of the leading and trailing leg markers directly above the highest point of the obstacle and subtracting the different heights of small- and large-stone obstacles, respectively (Figure 1). MFC was based either on toe or heel marker, whichever value was lower (Loverro et al., 2013). In addition, we computed crossing step length as the distance between the trailing toe and leading heel when crossing the obstacle (Buckley et al., 2010). Contrary to our initial intention, we did not compute the kinematic parameters relative to the baseline condition (by subtracting the relevant parameter of the smooth stimuli from the corresponding stone-covered surfaces) as our analysis revealed that unexpectedly foot clearance (especially for the leading foot) varied strongly for the two smooth stimuli but, in contrast, remained remarkably constant for the stone-covered surfaces (see Supplemental Figure S1). Specifically, lead MFC for the smooth-large stimulus was higher than clearance for all stone-covered stimuli, while lead MFC for the smooth-small stimulus was lower than clearance for all stone-covered stimuli. Thus, subtraction of the smooth stimuli would have resulted in clearance values with opposite signs (i.e., negative vs. positive).

To avoid this, we instead followed the more commonly used procedure of normalising values to each participant's leg length to account for interindividual differences. We analysed both normalised and nonnormalised data. As result patterns were similar for both variables, all reported results of models and correlations, tables, and figures in this paper are based on normalised data. Given that normalised data are in arbitrary units, we also show clearance and crossing step length in millimetre on the secondary *y*-axes of all figures to aid interpretation. The secondary *y*-axes were computed by multiplying the normalised values with the mean leg length of participants (see the Participants section). All data can be found on OSF (https://osf.io/mpk93/).

Before analysing each variable, outliers were checked and excluded as recommended by Chau et al. (2005) based on the boxplot method (1.5 × interquartile range) across participants. We analysed the data with linear mixed models (LMMs). As recommended by Barr et al. (2013), we opted for maximal models justified by design. Where the model failed to converge or showed a singular fit, the random structure was simplified iteratively, starting from the most complex structure. To determine the effect of perceived unpleasantness on stepping, we ranked the obstacles based on each participant's unpleasantness and roughness ratings and used these rankings as a fixed effect in the model (Model 1). Obstacles rated as the most unpleasant were ranked as 1, the next as 2, and so on. In cases where participants gave equal ratings to obstacles (e.g., two obstacles rated as 100), those obstacles were assigned the minimum rank (i.e., both would be ranked as 1). Given that we obtained ratings for four obstacles, the highest ranking would be 4. Additionally, informal chats and an open-ended question (see Supplemental Figure S2) suggested that some participants perceived the surfaces of the obstacle to be covered with softer materials (e.g., rubber) rather than stones. Therefore, we also explored whether MFC or crossing step length varied depending on hardness rankings using the same procedure. We also tested the effects of visual uncertainty in Model 1. Finally, while we controlled for the effects of trials in Model 1 by including them as fixed effects, we refrained from interpreting their main effects or interactions, as trials of smooth obstacles were excluded in this analysis. To summarise, we tested nine different variants of Model 1 with the formula: lead MFC/trail MFC/crossing step length ∼ 1 + trials + unpleasantness/roughness/hardness * visual uncertainty + (1 + visual uncertainty * unpleasantness/roughness/hardness||participants). The independent factors were the rankings of the perceptual ratings (unpleasantness, roughness, and hardness, respectively) and visual uncertainty, and the dependent variables were lead MFC, trail MFC, and crossing step length. To determine the effects of trials, we constructed a second model where we tested the effects of obstacle types (six unique combinations of stone size and density), visual uncertainty (binocular vs. monocular), trials, and their interactions (Model 2) on our three dependent variables (lead MFC, trail MFC, and crossing step length) with the formula: lead MFC/trail MFC/crossing step length ∼ 1 + trials * obstacle type * visual uncertainty + (1 + visual uncertainty * obstacle type||participants). In case of singular fit for both Models 1 and 2, interaction effects were removed from the random structure. Models were compared with the likelihood ratio test, the Akaike information criterion (AIC), and the Bayesian information criterion (BIC). Models with a lower AIC and BIC were preferred. The significance threshold was set to *p* < .05. We first checked whether our variables differed by whether participants started stepping with binocular or monocular vision first (i.e., block order). Block order did not affect any of the variables and was not considered in all models below. Correlations were tested with Pearson's correlation.

### Results

#### Perceptual Ratings

**Figure 2.**
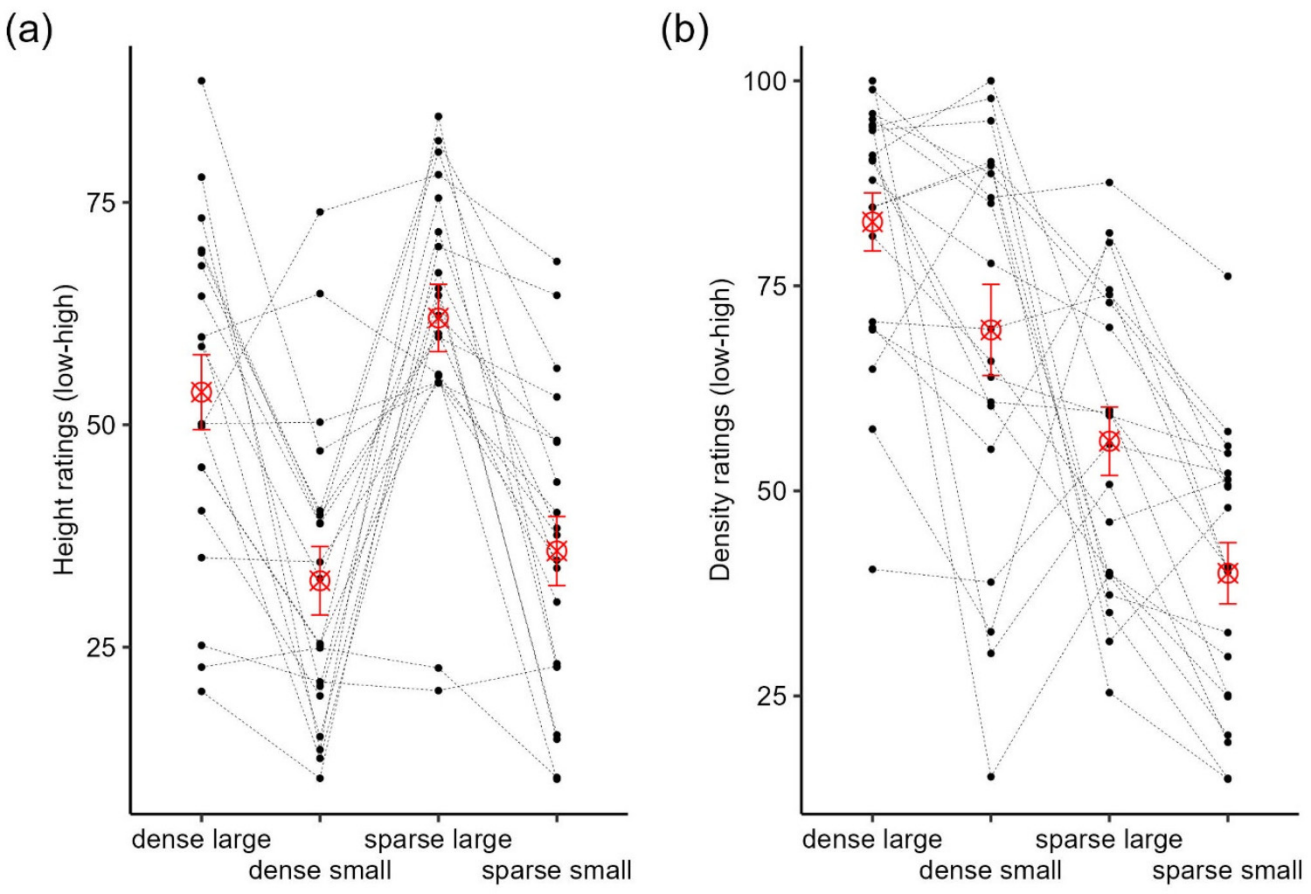
Ratings of (a) height and (b) density for each type of obstacle. Black dots and dotted lines represent individual participants, red ⊗ represents the mean ratings, and the error bars represent ±1 SEM.

First, we checked if our surface manipulations in height and density affected participants’ perceptions of physical attributes in the expected way with a 2 (stone size: small vs. large) × 2 (density: dense vs. sparse) repeated-measures ANOVA. Regarding ratings of surface height, as expected, there was a main effect of stone size, *F*(1, 19) = 35.61, *p* < .001, *ηp*^2^ = .65, but no significant main effect of density, *F*(1, 19) = 3.03, *p* = .10, *ηp*^2^ = .14. There was also no significant interaction between stone size and density on height ratings, *F*(1, 19) = 1.33, *p* = .26, *ηp*^2^ = .07. As can be seen in Figure 2a, the two obstacles covered with large stones received higher height ratings than the two obstacles covered with small stones independent of their density. These results suggest that the perceived height of obstacles was solely determined by stone size. Regarding ratings of density, there was a main effect of stone size, *F*(1, 19) = 19.54, *p* < .001, *ηp*^2^ = .51, and a main effect of density, *F*(1, 19) = 25.75, *p* < .001, *ηp*^2^ = .58. There was no significant interaction between stone size and density on density ratings, *F*(1, 19) = .23, *p* = .64, *ηp*^2^ = .01. That is, density ratings were dependent on both density and stone size with smaller stones being rated as less dense than large stones. As coverage in the sparse condition differed for large (20%) and small stones (10%), these ratings are consistent with the differences in coverage for large and small stone obstacles.

**Figure 3.**
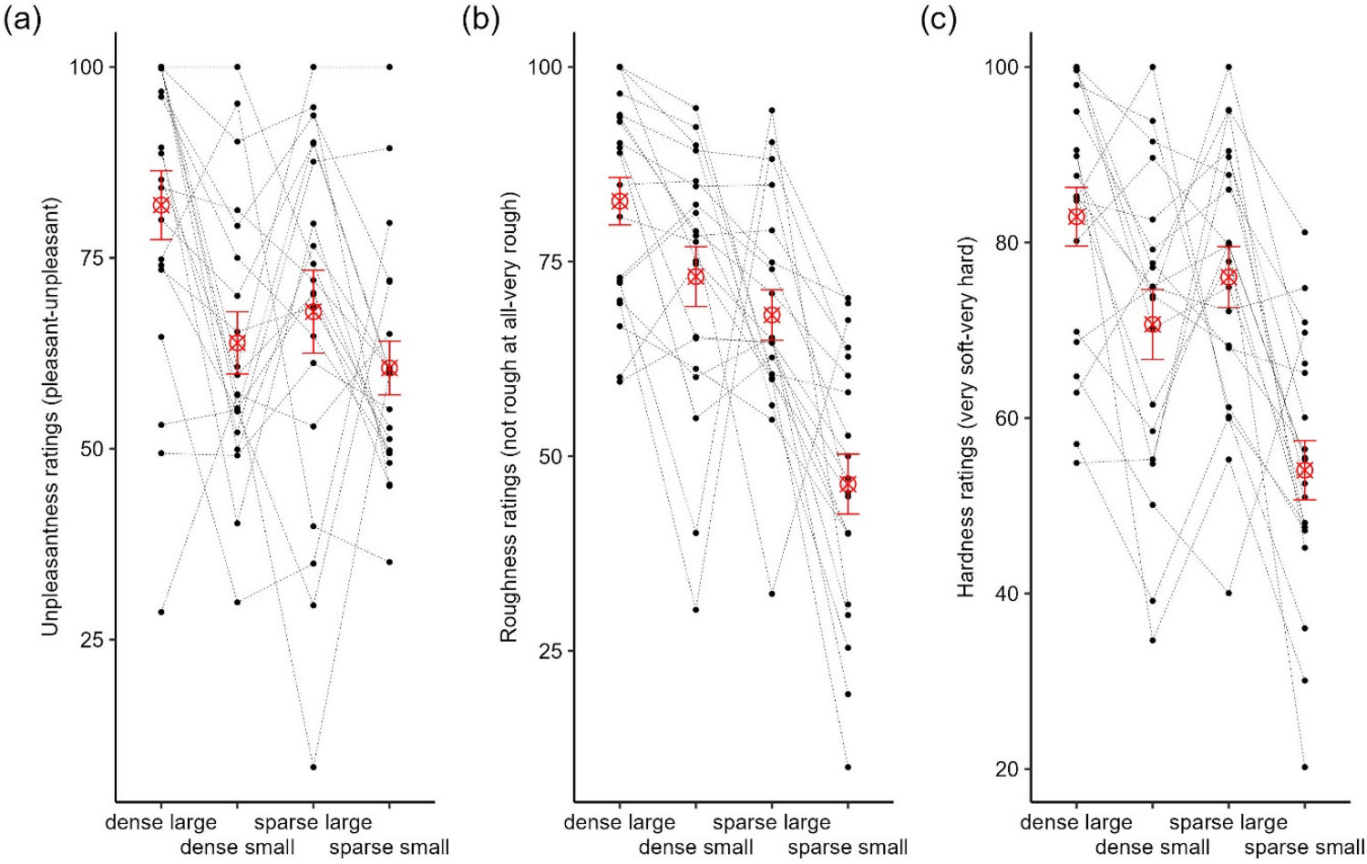
Ratings of (a) unpleasantness, (b) roughness, and (c) hardness for each type of obstacle. Black dots and dotted lines represent individual participants, red ⊗ represents the mean ratings, and the error bars represent ±1 SEM.

Regarding the ratings of roughness, hardness, and unpleasantness, the dense-large obstacle was, on average, rated significantly higher in unpleasantness (all *p*s < .03), roughness (all *p*s < .02), and hardness (all *p*s < .02 except for the comparison between dense-large and sparse-large) than all the other obstacles (Figure 3a–c). The sparse-small obstacle, on the other hand, was always rated as the least rough and least hard compared to the other obstacles. Importantly, as shown in Figure 3, perceived unpleasantness (and also perceived roughness and hardness) varied considerably across individuals for each obstacle, supporting our decision to rank obstacles based on individual's perceptual ratings in Model 1. Unpleasantness ratings were significantly correlated with all other perceptual ratings (.27 ≤ *r* ≤ .58). Interestingly, the highest correlation was between perceived unpleasantness and hardness (*r* = .58), suggesting that perceived unpleasantness was more strongly linked to perceived hardness than roughness (*r* = .42). Other perceptual ratings were significantly correlated with each other as well (see Table 1).

**Table 1.** Correlations between perceptual ratings in Experiments 1 and 2.

Experiment 1	1	2	3	4	5
1. Unpleasantness	1.00				
2. Roughness	.41***	1.00			
3. Hardness	.58***	.65***	1.00		
4. Height	.42***	.36**	.43***	1.00	
5. Density	.27*	.60***	.49***	.22*	1.00
Experiment 2	1	2	3	4	5
1. Unpleasantness	1.00				
2. Roughness	.76***	1.00			
3. Hardness	.79***	.78***	1.00		
4. Dangerousness	.81***	.83***	.85***	1.00	
5. Painfulness	.83***	.82***	.88***	.94***	1.00

Note that we added ratings of dangerousness and painfulness in Experiment 2.

*Note*. *p < .05, **p < .01, ***p < .001.

##### Model 1: Effects of Perceptual Ratings and Visual Uncertainty on Stepping Kinematics

**Figure 4.**
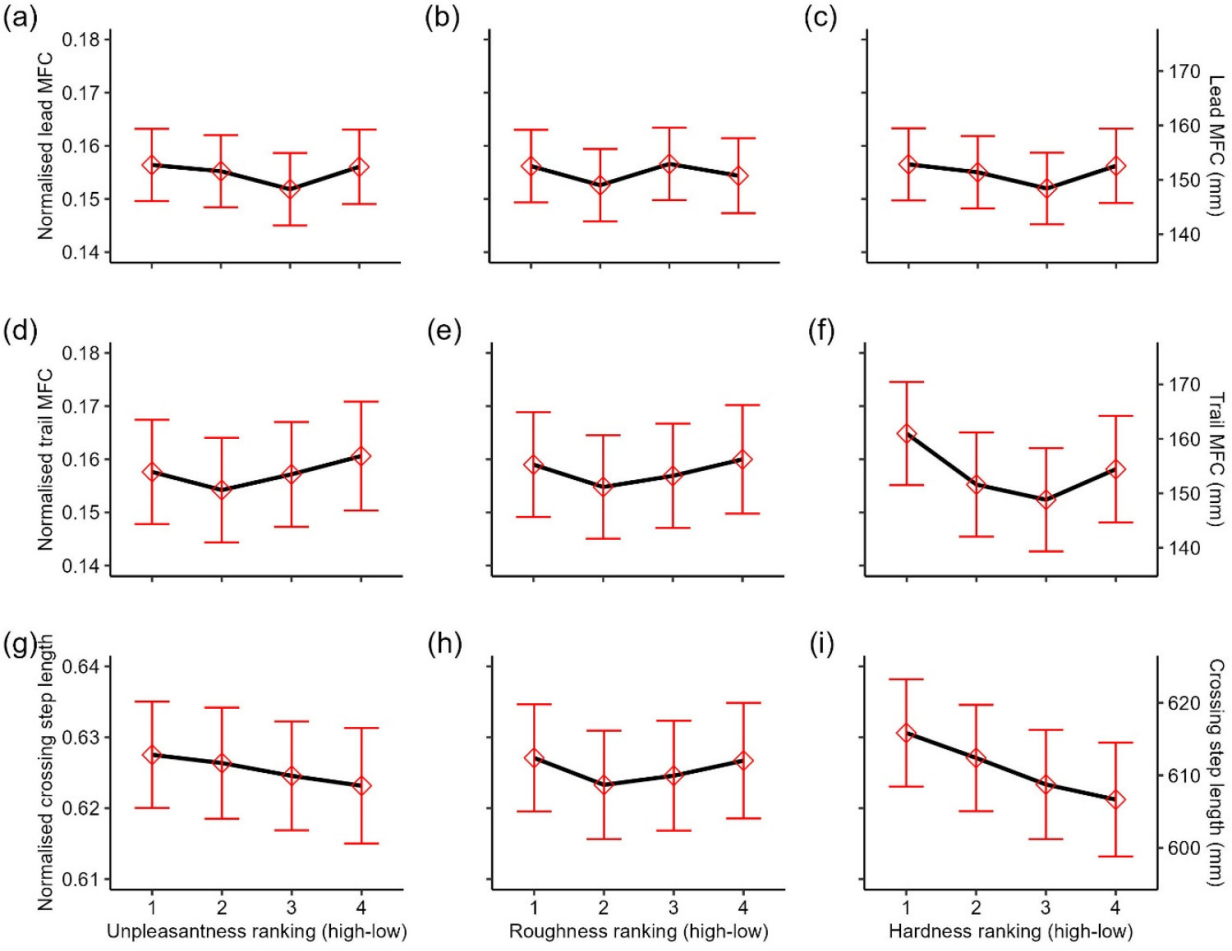
Estimated marginal means (the red open diamonds) of lead MFC, trail MFC, and crossing step length by unpleasantness (a, d, g), roughness (b, e, h), and hardness (c, f, i) rankings. Error bars represent ±1 SEM. Secondary *y*-axes were computed by multiplying the primary *y*-axes with the mean leg length of participants.

*Minimum foot clearance*. Lead MFC mainly came from the toe marker (67.4%), and all trail MFC came from the toe marker. A total of 15 (0.4%) leading leg observations and 173 (4.9%) trailing leg observations were excluded because they were identified as outliers. Descriptively, there seemed to be no linear trend in lead and trail MFC as the perceived unpleasantness, roughness, or hardness decreased (Figure 4a–f). We found no significant effects of perceived unpleasantness (Figure 4a), roughness (Figure 4b), and hardness (Figure 4c) on lead MFC (all *p*s > .08). Similarly, there were no significant effects of perceived unpleasantness (Figure 4d) and roughness (Figure 4e) on trail MFC (all *p*s > .54). However, there was a significant main effect of perceived hardness on trail MFC, *F*(3, 27.31) = 6.33, *p* = .002. Trail MFC over the obstacle with the highest perceived hardness in relation to the rest of the rankings appeared to be the primary driver of this effect (Figure 4f).

There was no significant main effect of visual uncertainty on lead MFC (all *p*s > .13) and trail MFC (all *p*s > .49). We found no significant interactions between visual uncertainty and perceived unpleasantness, roughness, and hardness on lead MFC (all *p*s > .25). Similarly, there were no significant interactions between visual uncertainty and perceived unpleasantness, roughness, and hardness on trail MFC (all *p*s > .28).

*Crossing step length*. Computation of crossing step length returned NAs in 25 trials (0.7%) due to poor data quality in either the trailing toe or leading heel. In addition, a total of 75 (2.1%) observations were excluded because they were identified as outliers. Descriptively, crossing step length tended to decrease almost linearly with a decrease in unpleasantness (Figure 4g) and hardness (Figure 4i), but did not show this trend for perceived roughness (Figure 4h) of the stimuli. However, these effects were not statistically significant (all *p*s > .11).

There was no significant main effect of visual uncertainty on crossing step length (all *p*s > .41). We found no significant interaction between visual uncertainty and perceived unpleasantness, roughness, and hardness on crossing step length (all *p*s > .14).

##### Model 2: Effects of Obstacle Type, Visual Uncertainty, and Trials on Stepping Kinematics

**Figure 5.**
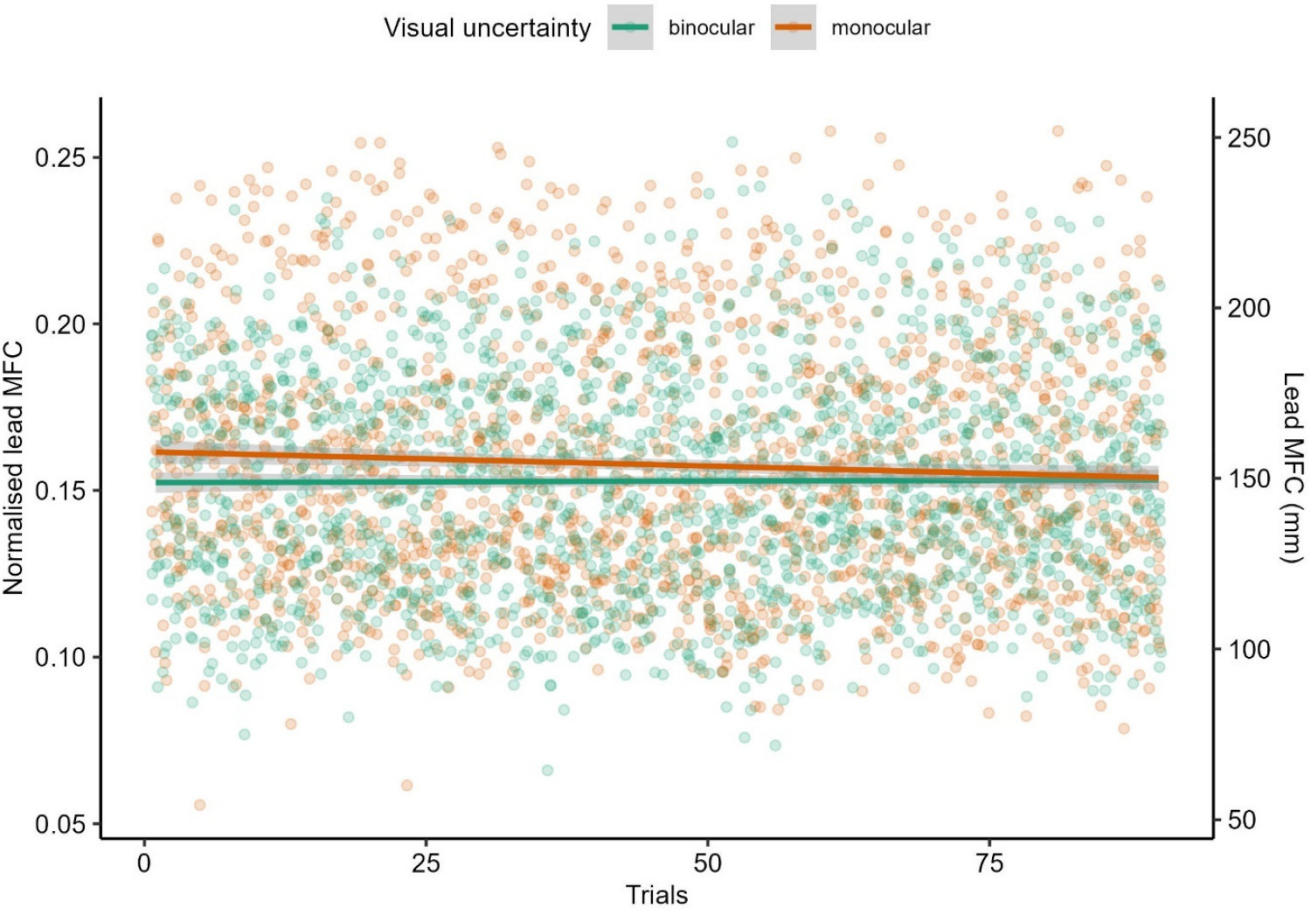
Interaction between visual uncertainty and trials on the lead MFC. Linear regression lines are fitted to trials by visual uncertainty. The shaded region represents the 95% confidence interval. Secondary *y*-axis was computed by multiplying the primary *y*-axis with the mean leg length of participants.

*Minimum foot clearance*. There were main effects of trials, *F*(1, 176.42) = 17.60, *p* < .001, obstacle type, *F*(5, 106.78) = 4.16, *p* = .002, and visual uncertainty, *F*(1, 36.48) = 7.66, *p* = .009, on lead MFC. The main effect of obstacle type was mainly driven by the higher lead MFC over the smooth-large obstacle compared to all the other obstacles (see Supplemental Figure S1). The main effects of trials and visual uncertainty were further qualified by a significant two-way interaction, *F*(1, 221.78) = 6.28, *p* = .013. Specifically, lead MFC was higher in the monocular condition than the binocular condition initially but decreased to the binocular level over trials (Figure 5). The reason why we found a main effect of visual uncertainty in Model 2 but not in Model 1 is likely due to increased power in detecting an effect (all trials were considered in Model 2). All other interactions were not significant (all *p*s > .30). For the trailing leg, there were no significant main effects of obstacle type, visual uncertainty, and trials or interactions between those factors (all *p*s > .06).

*Crossing step length*. We found a main effect of obstacle type, *F*(5, 115.88) = 5.05, *p* < .001, and a main effect of trials, *F*(1, 83.02) = 7.96, *p* = .006. They were further qualified by a significant interaction, *F*(5, 104.74) = 3.13, *p* = .011. However, because the conditions were not fully crossed due to incomplete randomisation, we believed that this two-way interaction might be unreliable. Ignoring the interaction, crossing step length tended to increase over trials (see Supplemental Figure S3a). There were no main effects of visual uncertainty, *F*(1, 45.05) = .89, *p* = .35, on crossing step length. All other interactions were not significant (all *p*s > .14).

##### Correlations Between Stepping Kinematics and Ratings

Since the obstacles differed across nearly all perceptual ratings, we were unable to determine whether perceived unpleasantness specifically influenced kinematics. Given the moderate and statistically significant correlations among perceptual ratings (Table 1), we conducted partial correlations to isolate the unique contribution of each perceptual property to kinematics. This approach allowed us to account for shared variance between ratings and better identify which perceptual properties were most strongly associated with kinematic adjustments.

*Minimum foot clearance*. As expected, based on our findings of Model 1 (Figure 4a–f), lead and trail MFC did not correlate with any of the perceptual ratings (all *p*s > .09; Table 2).

**Table 2.** Partial and Pearson (in parentheses) correlations between kinematics and a perceptual rating while controlling for all the other perceptual ratings.

Experiment 1	Lead MFC	Trail MFC	Crossing step length
Unpleasantness	.13 (.18)	.08 (.09)	.25* (.26*)
Roughness	−.05 (.06)	−.19 (−.09)	−.13 (−.02)
Hardness	.07 (.14)	−.01 (.02)	−.02 (.08)
Height	−.04 (.05)	.17 (.16)	.06 (.13)
Density	.03 (.07)	.13 (.05)	.03 (.02)
Experiment 2			
Unpleasantness	−.03 (.18)	−.39* (−.01)	−.02 (.01)
Roughness	−.19 (.13)	−.01 (.17)	−.28 (−.07)
Hardness	.09 (.23)	.13 (.22)	.01 (.01)
Dangerousness	.30 (.29*)	.26 (.27)	.42^**^ (.14)
Painfulness	−.14 (.22)	−.03 (.21)	−.25 (.01)

*Note*. *p < .05, **p < .01.

*Crossing step length*. A significant correlation was found between crossing step length and perceived unpleasantness, *r*(74) = .25, *p* = .03, while controlling for all other perceptual ratings. This is in line with the linear trend seen in Figure 4g based on the ranking data. That is, crossing step length increased for obstacles rated as more unpleasant. While there was a similar linear trend for crossing step length based on perceived hardness in the ranking data, the correlation between them based on continuous data did not reach statistical significance (Table 2). There was no significant correlation between perceived roughness and crossing step length.

### Discussion

Based on previous studies that observed that increased costs of action mistakes (such as stepping over fragile obstacles) result in an increased avoidance response (e.g., Patla et al., 1996), we hypothesised that unpleasantness may have similar effects on stepping kinematics. However, in this experiment, we did not find compelling evidence that stepping kinematics, specifically MFC, and crossing step length increase with the perceived unpleasantness of obstacle surfaces. There may be a couple of reasons for the lack of effects. Firstly, the obstacle height used by Patla et al. (1996) was considerably higher (26.8 cm) than in our study (∼9.3–9.9 cm). It is therefore possible that our obstacles were not challenging enough to reveal effects of perceived unpleasantness. This may also be the reason why, in contrast to previous studies (e.g., Heijnen et al., 2012), we did not find a decrease of MFC over trials in binocular vision (i.e., floor effect). Secondly, the variations we introduced in surface properties may have been too subtle to evoke reliable effects on stepping kinematics. This is supported by the weak but significant correlation we observed between crossing step length and perceived unpleasantness when considering the continuous rating data. Thirdly, continuously walking over the same obstacle may have further reduced kinematic variations by decreasing uncertainty.

Finally, the absence of an interaction between visual uncertainty and perceptual affective ratings in all stepping parameters ostensibly suggests that visual uncertainty did not significantly alter how obstacles were perceived or approached during the study. However, alternatively, another reason for this could be that while monocular vision may have resulted in a decrease in the perceived roughness of the obstacles (as observed by Allison et al., 2009), which should result in lower clearance levels, the increased visual uncertainty may have the opposite effect of increasing overall clearance levels in monocular stepping. Thus, both effects may cancel each other, resulting in the observed null findings. As we did not collect surface ratings in a monocular viewing condition, this remains speculative. Since the stepping task always preceded the rating task, participants had already seen all obstacles under both binocular and monocular conditions. As a result, any ratings based solely on monocular viewing would likely be unreliable due to prior binocular exposure. Furthermore, we did also not ask for ratings of the smooth surfaces in Experiment 1, and this prevented us from ranking smooth obstacles and comparing effects across all obstacles. The reasoning for this omission was that we were concerned how our participants would rate the density of smooth surfaces.

To address these concerns, we conducted a second experiment (Experiment 2) in which we used three different, and more challenging, obstacle heights (6.1, 12.1, and 18.1 cm), introduced a more prominent variation of surface texture (i.e., spiky vs. smooth), assessed stepping in discrete trials, and asked for ratings of the smooth obstacle. The type of surface variations (i.e., roughness), we introduced to manipulate unpleasantness in this study, is likely also associated with dangerousness and/or painfulness – aspects we did not assess in Experiment 1. Increasing the differences in surface texture will increase the likelihood that obstacles may be perceived as dangerous and/or painful to step on rather than just as unpleasant. To be able to disentangle effects of unpleasantness from those of dangerousness and painfulness, we added perceptual ratings assessing those aspects in Experiment 2.

## Experiment 2

### Method

#### Participants

Twenty-four naïve participants were recruited. Two participants were excluded from all analyses (one due to poor data quality and another due to misunderstanding the rating task). Hence, the following results were based on 22 participants (17 females, 4 males, and 1 self-identified as other) aged between 18 and 39 years (*M* = 22.23, *SD* = 4.79) with a mean body height of 168.7 cm (*SD* = 8.51) and a mean leg length of 93.8 cm (*SD* = 5.24). All participants were able to walk over the obstacles of different heights without difficulties and had normal or corrected-to-normal visual acuity. This study complied with the Ethics Code of the British Psychological Society (BPS) and was approved by the Psychology Ethics Committee of the University of Aberdeen (ID: 694797). Participants provided written informed consent before the experiment and were reimbursed either with course credits or £8/hour upon completion.

#### Stimuli and Apparatus

**Figure 6.**
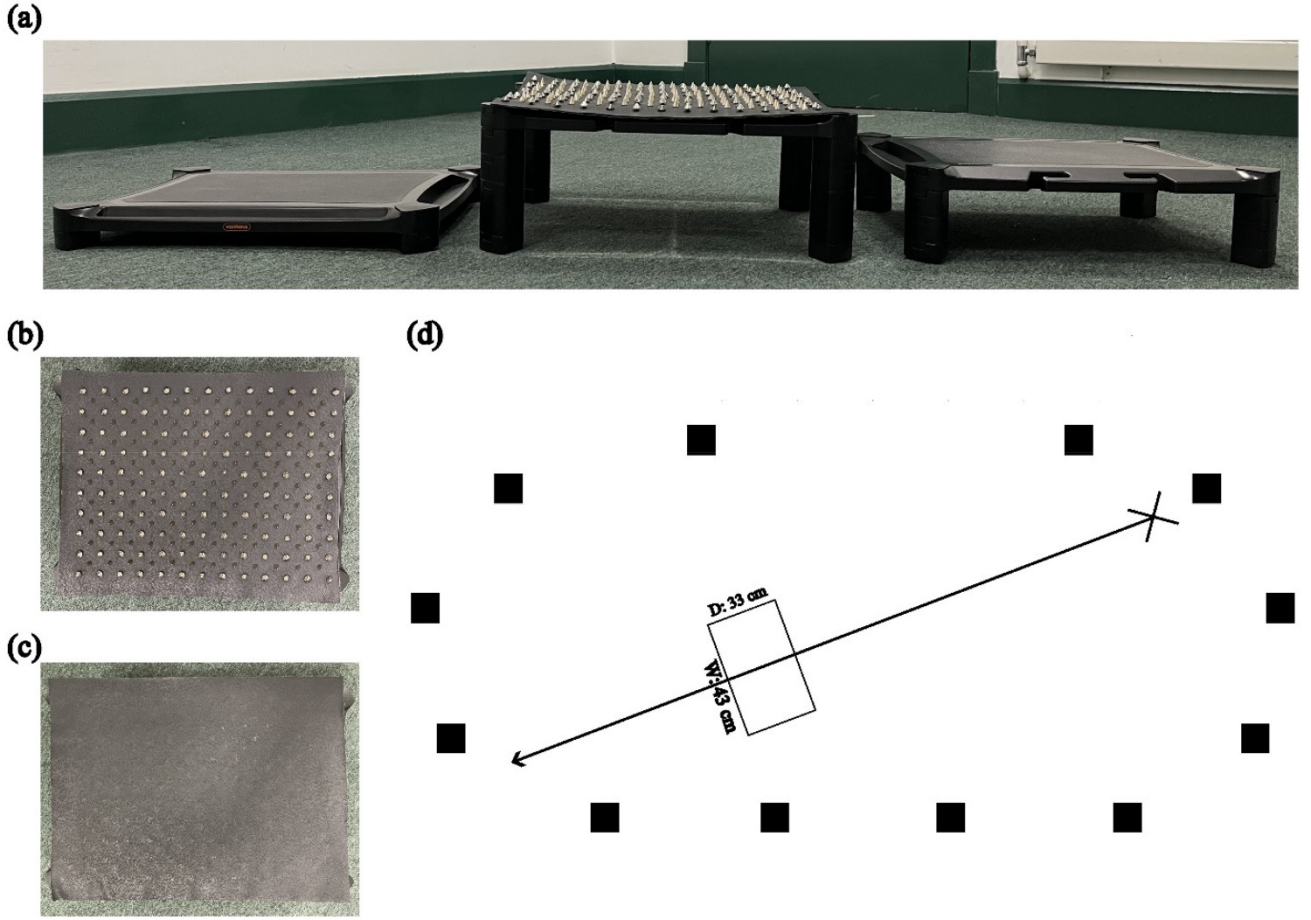
(a) Obstacles of the three different heights with the highest one covered with the spiky surface. Top view of the (b) spiky and (c) smooth obstacle surface. (d) Top view of the experimental layout. The cross represents the starting position, the rectangle represents the obstacle, and the black boxes represent the cameras. The figure is not drawn to scale.

The obstacles were three height adjustable monitor stands (width = 43 cm, depth = 33 cm, heights = 5, 11, and 17 cm; Figure 6a). The surface of the monitor stand was covered with a black leather fabric covered with metal spike studs (7 × 11 mm). The black leather fabric was covered with evenly distributed metal spike studs (3 cm between adjacent horizontal and vertical spikes and 2.5 cm between diagonal spikes). For the spiky surface, the leather fabric was placed on top of the monitor stand with the spikes facing upward (Figure 6b). For the smooth surface, the leather fabric was place with the spikes facing downward (Figure 6c). The final heights of the obstacles, considering the heights of the leather fabric and spikes, were 6.1, 12.1, and 18.1 cm, and they will be referred to as h1, h2, and h3, respectively. Arrangements of markers and motion tracking cameras were identical to Experiment 1 (see Figures 1c, 1d and 6d).

#### Procedure

Similar to Experiment 1, Experiment 2 also consisted of a stepping (obstacle avoidance) task and a rating task. The stepping task always preceded the rating task. The stepping task consisted of two blocks: in one block, participants stepped over the spiky obstacles, and in the other block, over the smooth obstacles. We counterbalanced the blocks so that half of the participants stepped over the spiky obstacles first and the other half stepped over the smooth obstacles first. Heights of obstacles were presented randomly within each block. Each block contained 30 trials, with 10 trials for each obstacle height.

Before starting the stepping task, participants were asked to stand in a fixed position that was about 2.2 m away from the obstacle. In this experiment, instead of walking continuously, participants stepped over the obstacle and stopped at a specified position (∼1.25 m after the obstacle) using a straight walking path (Figure 6d). Three successive beep sounds were played to signal the participants to start walking. At the end of the path, a beep sound was played to signal participants to stop and walk back to the starting position. We instructed participants to walk as naturally as possible at a self-selected pace.

For the rating task, participants were instructed to sit on a chair at the starting position of the stepping task and visually rate the obstacle's surface that was placed in the same position as in the stepping task on perceived unpleasantness, roughness, hardness, dangerousness, and painfulness by pointing with a mouse on a continuous scale between 0 and 100. Specifically, we asked participants to imagine how unpleasant, dangerous, and painful it would be to step on the surface of the obstacle for the rating. The presentation of surface was randomised, and each surface was rated only once. There were no practice trials for both tasks, and the entire experimental sessions lasted for approximately 60 min.

#### Data Analysis

Kinematic data were filtered and extracted as in Experiment 1 (lead MFC, trail MFC, and crossing step length). However, it should be noted that we computed lead and trail MFC as the position of the markers when passing the front edge of the obstacle. This is because unlike the obstacles in Experiment 1, which had a highest point (see Figure 1), the obstacles in Experiment 2 were flat (see Figure 6). Out of 1320 trials from 22 participants, only two trials were excluded due to obstruction of markers during data collection. We first checked whether our variables differed by whether participants stepped over the smooth or the spiky surface first (i.e., block order). Block order did not affect any of the variables and was not considered in the models below.

All variables were normalised to participants’ leg length and analysed in the same way as in Experiment 1. Specifically, all models tested the main effects of trials (30 trials), obstacle height (h1, h2, h3), obstacle surface (smooth and spiky), and their interactions with the formula (lead MFC/trail MFC/crossing step length ∼ 1 + trials * height * surface + (1 + height + obstacle surface|participants). Interaction effects were not tested in the random structure due to singular fit.

### Results

#### Perceptual Ratings

**Figure 7.**
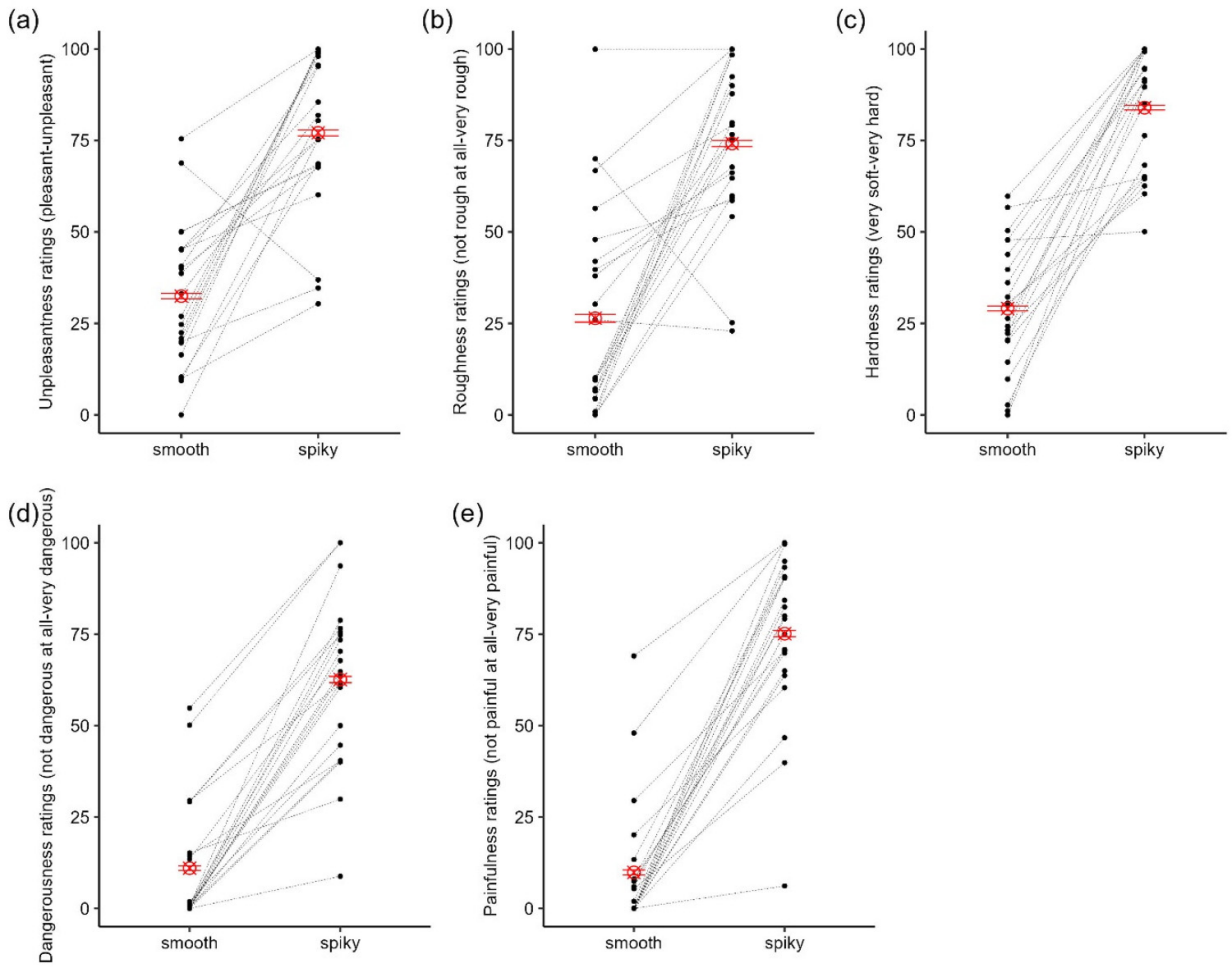
Ratings of (a) unpleasantness, (b) roughness, (c) hardness, (d) dangerousness, and (e) painfulness for smooth and spiky obstacles. Black dots and dotted lines represent individual participants, red ⊗ represents the mean ratings, and the error bars represent ±1 SEM.

The spiky obstacle was rated (*M* = 77.1, *SD* = 21.3), on average, as more unpleasant than the smooth obstacle (*M* = 32.4, *SD* = 19.0), *t*(21) = −7.00, *p* < .001 (Figure 7a). The spiky obstacle was also rated, on average, as rougher, harder, more dangerous, and more painful when stepped on, than the smooth obstacle (all *p*s < .001, Figure 7b–e). All perceptual ratings were moderately to highly correlated (.76 ≤ *r* ≤ .94; see Table 1). Two participants rated the smooth obstacle as more unpleasant or rougher than the spiky obstacle, respectively (see Figure 7a and b). However, since the majority of the participants rated the spiky obstacle as more unpleasant, rougher, harder, dangerous, and painful than the smooth obstacle, we believed there was no need to rank obstacles based on each participant's ratings as was done in Experiment 1.

#### Effects of Obstacle Height, Obstacle Surface, and Trials on Stepping Kinematics

**Figure 8.**
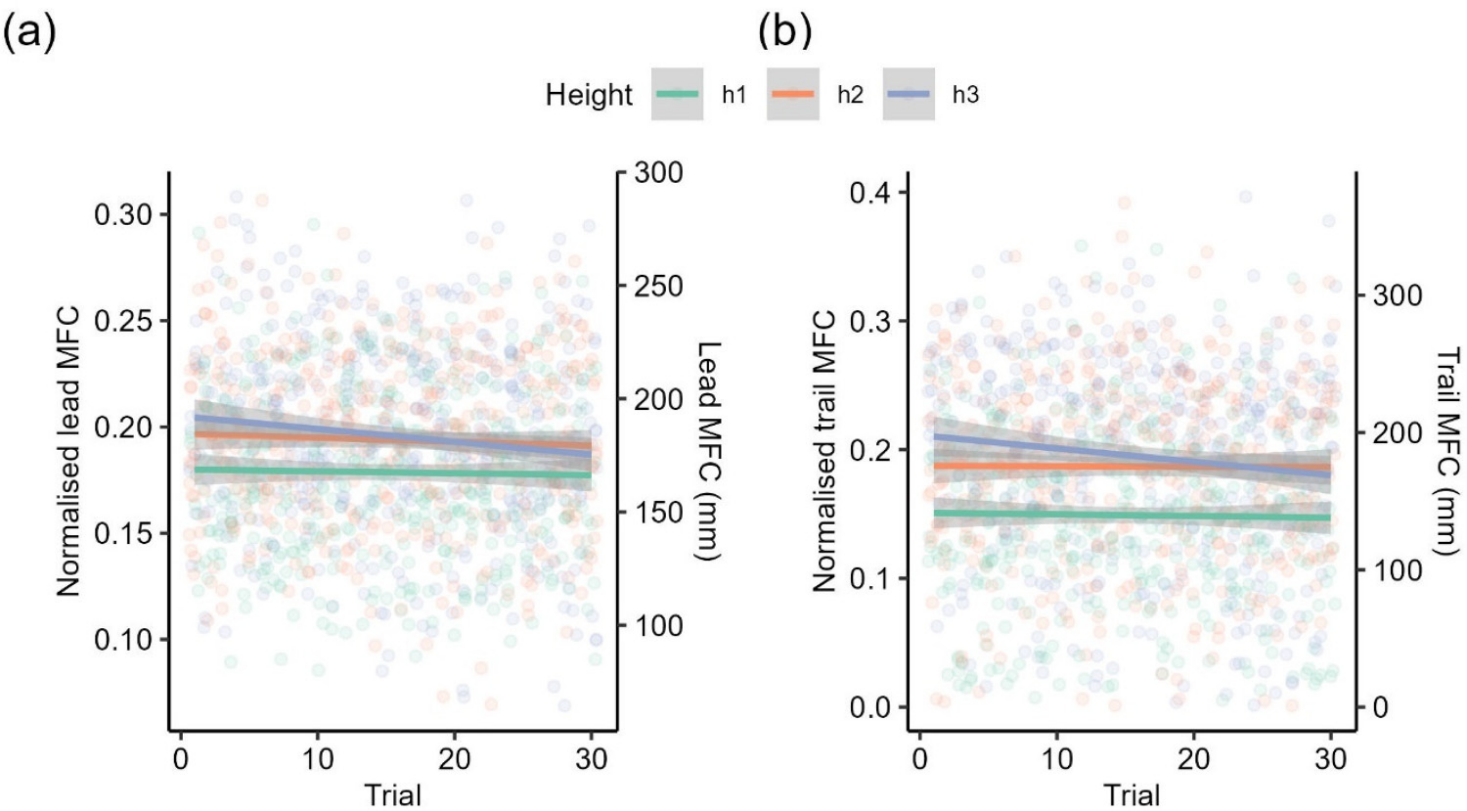
(a) Lead and (b) trail MFC by different obstacle heights over trials. Linear regression lines are fitted to trials by different obstacle heights. The shaded region represents the 95% confidence interval. Dots represent individual observations. Secondary *y*-axes were computed by multiplying the primary *y*-axis with the mean leg length of participants.

**Figure 9.**
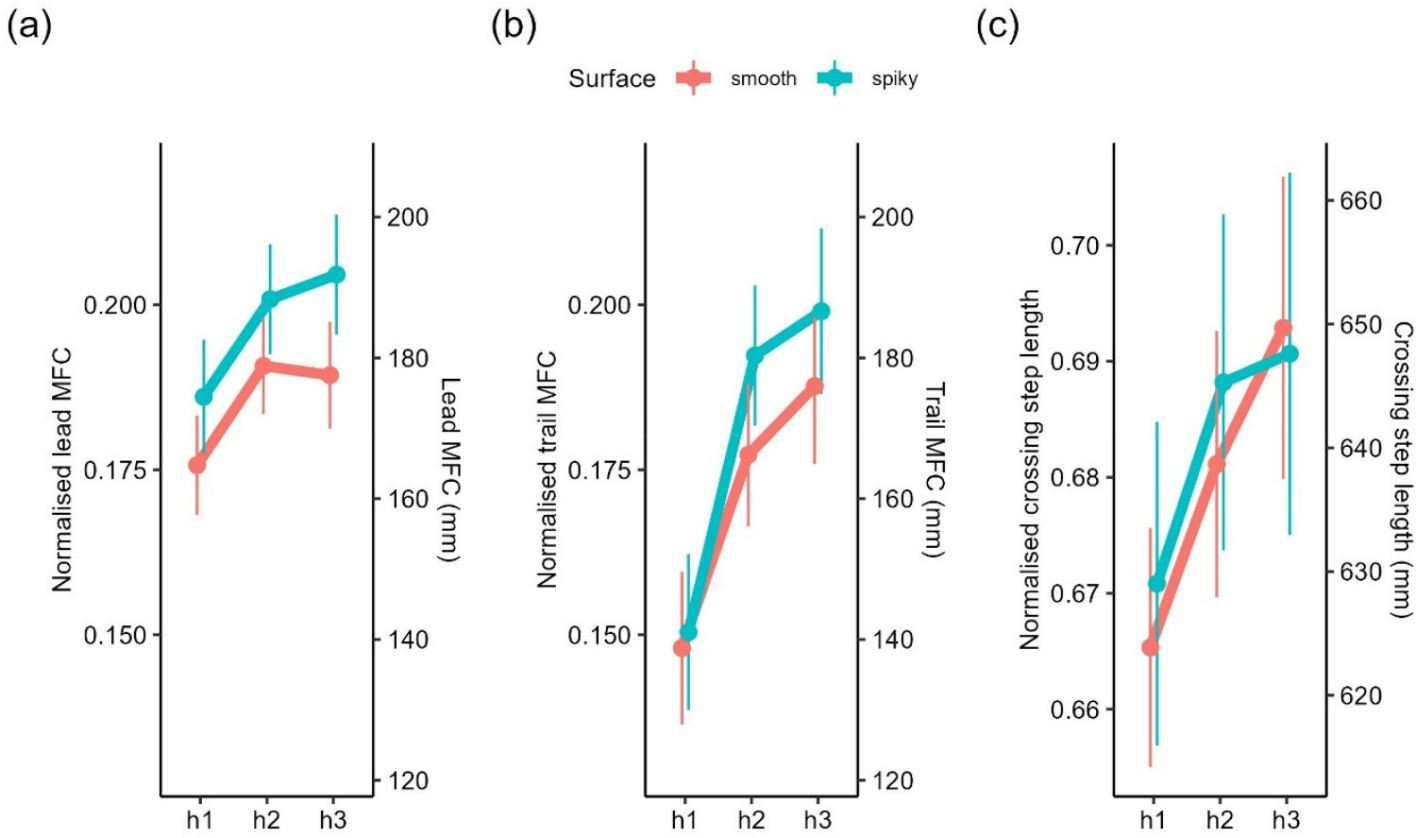
Estimated marginal means of (a) lead, (b) trail MFC, and (c) crossing step length over different obstacle heights by surface. Error bars represent ±1 SEM. Secondary *y*-axes were computed by multiplying the primary *y*-axes with the mean leg length of participants.

*Minimum foot clearance*. Lead MFC mainly came from the toe marker (99%), and all trail MFC came from the toe marker. Computation of two (0.2%) leading leg observations and 64 (4.9%) trailing leg observations returned NAs due to poor data quality or the presence of implausible negative values. In addition, a total of 28 (2.1%) leading leg observations and 18 (1.4%) trailing leg observations were excluded because they were identified as outliers. We found main effects of trials, *F*(1, 1217.12) = 12.74, *p* < .001, height, *F*(2, 79.34) = 13.22, *p* < .001, and surface, *F*(1, 34.09) = 8.63, *p* = .006, on lead MFC. The main effects of trials and height were further qualified by a significant interaction, *F*(2, 1218.32) = 3.67, *p* = .03, on lead MFC. Lead MFC was always higher for obstacles h2 and h3 compared to h1. Additionally, for obstacle h3, lead MFC gradually declined to the level h2 over trials (Figure 8a). The main effect of surface was driven by the higher lead MFC over the spiky obstacle compared to the smooth obstacle (Figure 9a). All other interactions were not significant (*p*s > .18). For trail MFC, there were main effects of height, *F*(2, 80.65) = 16.58, *p* < .001, and a marginally main effect of trials, *F*(1, 1165.47) = 3.54, *p* = .06. These main effects were further qualified by a significant interaction between trials and height, *F*(2, 1160.86) = 4.25, *p* = .014. The pattern was identical to lead MFC. Trail MFC was always higher for obstacles h2 and h3 compared to h1. Additionally, for obstacle h3, trail MFC gradually declined to the level of h2 over trials (Figure 8b). There were no significant main effects of surface, *F*(1, 39.79) = .28, *p* = .60, but there was a significant interaction between height and surface on trail MFC, *F*(2, 1160.98) = 3.16, *p* = .04. Trail MFC was similar over the obstacle at h1 regardless of the surface, but trail MFC was higher for the spiky surface than smooth surface over the obstacle at h2 and h3 (Figure 9b). All other interactions were not significant (*p*s > .17).

*Crossing step length*. Computation of crossing step length returned NAs in three trials (0.2%) due to poor data quality in either the trailing toe or leading heel. Three (0.2%) additional observations were excluded because they were identified as outliers. We found a main effect of trials, *F*(1, 1246.38) = 10.72, *p* = .001. Similar to Experiment 1, crossing step length increased over trials, with a mean difference of ∼18 mm between the first and last trial. Although crossing step length increased descriptively with height (Figure 9c), there were no main effects of height, *F*(2, 113.25) = 1.80, *p* = .17, and surface, *F*(1, 39.09) = .001, *p* = .95. Other main effects and interactions were not significant (all *p*s > .07).

#### Correlations Between Kinematics and Ratings

Since the spiky and smooth obstacles differed across all perceptual ratings (Figure 7), we were unable to determine whether perceived unpleasantness specifically drove the difference in lead and trail MFC between spiky and smooth obstacles. Moreover, there were high correlations among perceptual ratings (Table 1). To better isolate the unique contribution of each perceptual property to kinematics, we conducted partial correlations. This approach allowed us to account for shared variance between ratings and better identify which perceptual properties were most strongly associated with kinematic adjustments (Table 2).

Lead MFC was marginally correlated with perceived dangerousness, *r*(38) = .30, *p* = .06. As perceived dangerousness increased, lead MFC increased. Similarly, crossing step length correlated moderately with dangerousness with crossing step length increasing as perceived dangerousness increased, *r*(38) = .42, *p* = .006. Unexpectedly, and counterintuitively, we also observed a negative correlation between trail MFC and perceived unpleasantness, *r*(38) = −.39, *p* = .01; as perceived unpleasantness increased, trail MFC decreased.

### Discussion

By making obstacles more challenging (i.e., increasing their height) and increasing the difference in surface texture, we found that lead and trail MFC increased for the spiky obstacle. Our rating data indicated that spiky obstacles were also perceived as more unpleasant to step on but also as more dangerous and painful. While this seems ostensibly in line with our original hypothesis that perceived unpleasantness increases the avoidance response, partial correlation isolating the unique contributions of the different perceptual aspects suggested that this effect is primarily driven by the perceived danger. Moreover, in line with previous findings (Heijnen et al., 2012), we found an effect of trials on both lead and trail MFC under binocular vision. These findings suggest that these effects may only become apparent when obstacles are sufficiently challenging. This could explain why we did not observe an effect of perceived unpleasantness and trials on MFC in Experiment 1.

While we tried to isolate the effects of unpleasantness from other associated factors such as roughness and hardness in Experiment 1, we did not include other closely related factors such as dangerousness or painfulness. To better disentangle the effects of unpleasantness from dangerousness and painfulness, we conducted partial correlations in Experiment 2, controlling for all factors closely related to unpleasantness. The results indicate that perceived unpleasantness per se did not drive the kinematic differences in Experiment 2. Instead, perceived dangerousness of stepping on the obstacle emerged as the primary factor, likely influencing participants’ responses to spiky versus smooth obstacles.

## General Discussion

This study aimed to explore if and how affective perceptual properties such as perceived unpleasantness of obstacles affect stepping movements. In addition, we examined the effects of perceptual and biomechanical task demands: in Experiment 1 by introducing visual uncertainty (binocular vs. monocular viewing) and in Experiment 2 by altering obstacle height.

### Effects of Affective Perceptual Ratings on Stepping Kinematics

We hypothesised that avoidance responses, measured as MFC and crossing step length, increase with perceived unpleasantness of an obstacle. In Experiment 1, we varied perceived unpleasantness by altering the roughness of the obstacles’ surface (using stones of varying size and density). When participants rated the perceived unpleasantness, roughness, and hardness of these surfaces, we found large individual differences in their perceptual judgments (Figure 3) – not only in the roughness and unpleasantness but also in hardness as participants perceived the stones as different materials such as rubber or even cardboard (Figure S2). To account for this, we used rankings of each participant’s perceptual judgements as the independent variable to determine the effect of affective obstacle properties on their stepping kinematics. Using this approach, we found no differences in lead and trail MFC between obstacles of varying perceived unpleasantness. Furthermore, unpleasantness ratings did not correlate with MFC, suggesting that clearance was not influenced by perceived unpleasantness. Crossing step length descriptively increased with higher unpleasantness ratings, but the effect was not statistically significant. However, the correlational analysis confirmed a weak but significant relationship between the continuous unpleasantness ratings and crossing step length. When transforming continuous ratings to ranking data, some information is inevitably lost, which could explain why we found no effects based on the ranking data but found significant correlations when using the continuous ratings. Potential reasons for the lack of effects in Experiment 1 may have been that (a) obstacles were not challenging enough (height < 10 cm) and (b) that surface variations did not elicit strong enough differences in perceived unpleasantness.

To address these limitations, we conducted a second experiment in which we introduced greater variation in obstacle height and a more distinct manipulation of surface properties (smooth vs. spiky). Participants’ perceptual ratings indicated that spiky surfaces were perceived relatively consistently as more unpleasant to step on but also as much more dangerous and painful. The effect of spikiness was also reflected in stepping kinematics with larger MFC for spiky obstacles. However, spikiness did not affect crossing step length. Examining the relationship between perceptual ratings and stepping adjustments, we observed a relationship between perceived dangerousness, but not unpleasantness, and crossing step length.

The shift from an unpleasantness-step length relationship in Experiment 1 to a dangerousness-step length relationship in Experiment 2 suggests that the nature of the obstacle may influence how participants categorised these obstacles. In Experiment 1, unpleasantness may have captured both discomfort and an implicit sense of risk, as we did not assess perceived dangerousness or painfulness. Still, if this was the case, then we would have predicted to find again a significant Pearson correlation between unpleasantness and crossing step length in Experiment 2 (which may, or may not, disappear if dangerousness is accounted for). However, this was not the case. At this point, we can only speculate about the reasons for this change. One possibility is that when obstacles are more hazardous, stepping adjustments may be primarily driven by their perceived threat rather than general discomfort.

The overall increase in MFC for spiky obstacles in Experiment 2 suggests that participants consistently lift their feet higher for obstacles that are more costly to collide with – regardless of interindividual differences in the subjective perceptual evaluation (i.e., rating) of the stimuli. Another potential explanation for the general higher MFC for spiky objects may be that participants simply overestimate their height compared to the smooth obstacles, leading to increased clearance. However, as we did not obtain height estimates in Experiment 2, this remains speculative. The fact that we did not observe an effect of obstacle surface on MFC in Experiment 1 is likely due to the overall smaller obstacle heights, differences in obstacle heights, and differences in surfaces variations.

Finally, crossing step length did not differ significantly between obstacle types (in both Experiments 1 and 2) but correlated with interindividual differences in perception. The fact that we observed correlations between perceptual ratings and crossing step length, but not MFC, may be due to our experimental design. In both experiments, obstacle height varied, while obstacle depth remained constant. Thus, participants may have been more concerned about tripping (which relates more to MFC) than stepping on the obstacles (which relates more to crossing step length), as height was the dimension of change (Begg et al., 2007). Because crossing step length was less mechanically constrained by obstacle height than MFC, participants had more flexibility in adjusting it based on their perception of obstacle properties. In contrast, MFC adjustments were likely dictated primarily by the obstacle's height, making it less sensitive to individual differences in perception. The overall increase in MFC for spiky obstacles suggests that participants adjusted their foot clearance in response to obstacle properties, but the lack of a correlation with perceptual ratings may reflect reduced variability due to biomechanical constraints (i.e., less manoeuvring room) rather than an absence of perceptual influence.

Together, these findings suggest that stepping adjustments are influenced by both physical properties of obstacles and individual differences in perceived cost of collision (Kangur et al., 2017; Miura et al., 2023; Patla et al., 1996; Rietdyk & Rhea, 2011).

### Effects of Visual Uncertainty, Obstacle Height, and Trials

In Experiment 1, we found that lead MFC differed between monocular and binocular viewing conditions corroborating the idea that binocular vision is critical to motor planning and execution in the upper and lower limbs (Cohen et al., 2010; Hayhoe et al., 2009; Melmoth & Grant, 2006; Patla et al., 2002; Schlicht & Schrater, 2007). Interestingly, we also found an interaction between visual uncertainty and trials in lead MFC. Specifically, with monocular vision, participants showed higher lead MFC in the early trials but slowly decreased to the level of clearance in binocular vision over trials. Trail MFC stayed relatively stable in binocular trials, which is in contrast to the findings of Heijnen et al. (2012), who observed a progressive decrease over trials in the MFC with binocular vision. A potential explanation is that their obstacles were overall more challenging in respect of height (ranging from 19.5 to 26.0 cm) than our obstacles (∼9.3–9.9 cm). Hence, in Heijnen et al.'s study, participants might have initially overshot their foot to ensure safe clearance but progressively decreased the foot clearance as they gained confidence over trials to reduce energy expenditure. This is further supported by our findings in Experiment 2, where we observed a decrease in both lead and trail MFC for the highest obstacle (to the level of the second highest obstacle) over trials. The fact that the trial-related effects on MFC only emerged under monocular vision (Experiment 1) and for the highest obstacle under binocular vision (Experiment 2) indeed suggest that the effects only occur when the task is sufficiently challenging. Overall, these findings suggest that safety margins are increased in earlier trials to compensate for increased uncertainty and/or task difficulty (Cohen et al., 2010; Heijnen et al., 2012; Sannier et al., 2023).

Importantly, our findings may help explain the mixed results in the literature regarding whether clearance of the leading and trailing foot increases with obstacle height. While some studies report an increase in clearance with height (Austin et al., 1999; Chen et al., 1991; Shin et al., 2015), others find stable clearance (Chou & Draganich, 1997; Kunimune & Okada, 2017) or even a decrease (Buckley et al., 2010; Kunimune & Okada, 2017). In general, our data suggest that both lead and trail MFC increase with obstacle height but show different trends across trials depending on the obstacle height (see Figure 8a and b). However, statistical approaches that rely on average clearance across multiple trials may overlook the decreasing trend of clearance over trials for challenging situations, potentially leading to conclusions of stable or decreasing clearance. In summary, our findings highlight the robust effects of trials and their interactions with visual uncertainty and obstacle height, which may help to explain the mixed results in the literature. This underscores the importance of accounting for trial effects and their interactions with independent variables in future research to gain a more accurate understanding of the data.

One limitation of our study concerns the quality of our kinematic data in Experiment 1, where a substantial number of participants had to be excluded from the analysis. This was primarily due to using the continuous stepping task, which did not allow us to exclude trials in which markers were not visible during data collection. Thus, if markers were not placed optimally at the start, or moved during the walking task, we had no opportunity to adjust them within a walking block of 15 trials. This was no longer the case in Experiment 2 where each step was collected as a single separate trial (and hence data quality improved considerably). However, despite this issue, we would argue that the consistency of our findings across both experiments, along with a good agreement with prior studies, speaks for the reliability of our data.

## Conclusion

This study provides preliminary evidence that affective perceptual properties such as dangerousness modulate avoidance kinematics in stepping. Similar to findings in other action tasks, our results suggest that adjustments are highly variable and strongly depend on specific stimulus characteristics and task demands (Cohen et al., 2010; Hesse et al., 2016; Sannier et al., 2023; Schenk, 2010). An issue may be that it might be hard, or even impossible, to isolate the effects of unpleasantness from those of other sensations such as pain, dangerousness, and disgust that may be actually driving the observed effects on actions and/or overwriting the effects of mere unpleasantness.

## Supplemental Material

sj-docx-1-pec-10.1177_03010066251360582 - Supplemental material for From discomfort to danger: Exploring how affective obstacle properties influence avoidance in stepping Supplemental material, sj-docx-1-pec-10.1177_03010066251360582 for From discomfort to danger: Exploring how affective obstacle properties influence avoidance in stepping by Zhong Jian Chee, Martin Giesel and Constanze Hesse in Perception
