# Supplementary material for: From discomfort to danger: Exploring how affective obstacle properties influence avoidance in stepping
Source: Perception. 2025 Jul 30;54(12):907–30. doi: 10.1177/03010066251360582 (PMC12605311; doi:10.1177/03010066251360582)
Supplement: sj-docx-1-pec-10.1177_03010066251360582 - Supplemental material for From discomfort to danger: Exploring how affective obstacle properties influence avoidance in stepping [file sj-docx-1-pec-10.1177_03010066251360582.docx]

**Figure S1**

(a) Lead MFC, (b) trail MFC, and (c) crossing step length by obstacle types. Error bars represent ± 1 SEM.

**
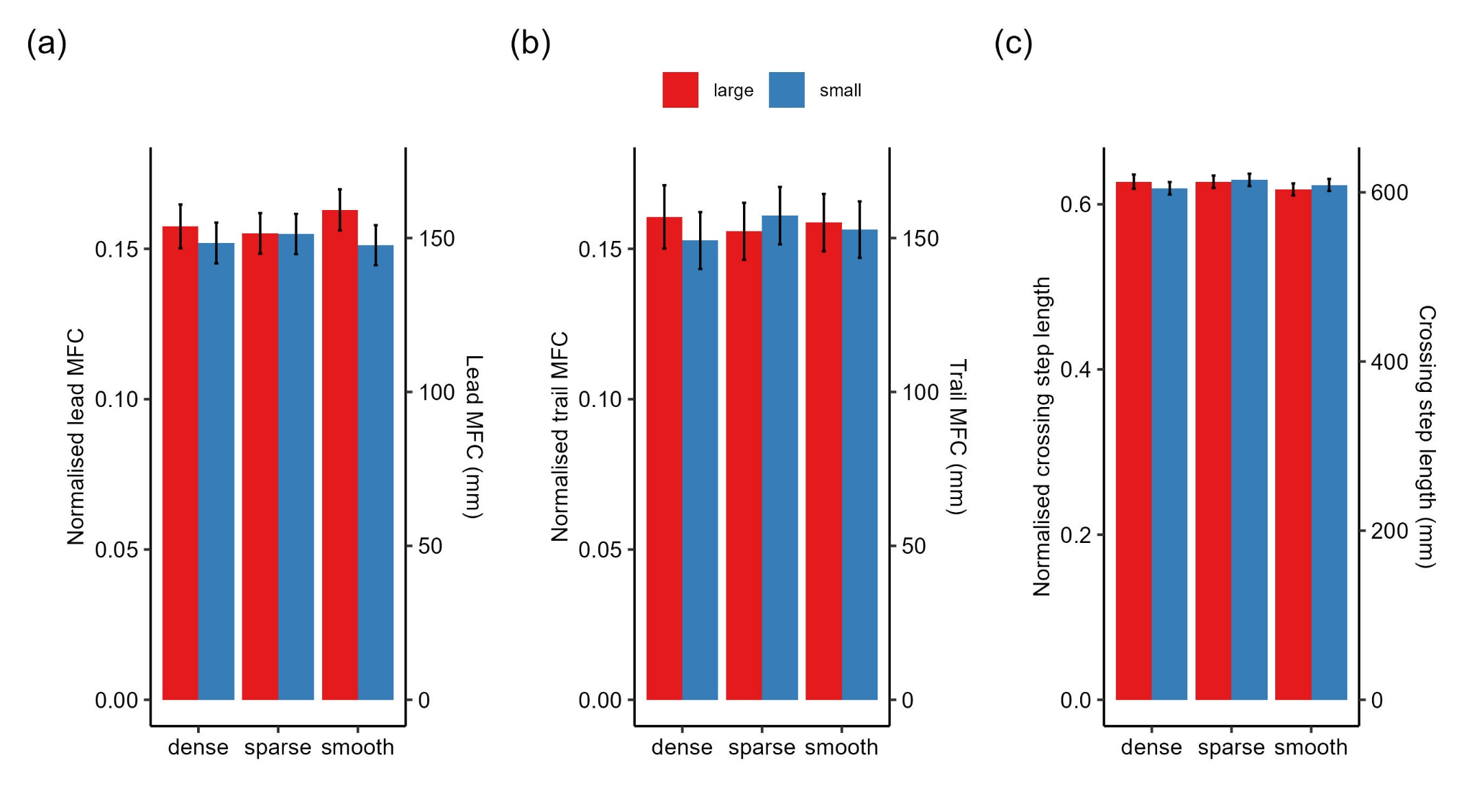
**

**Figure S2**

Participants’ label for each obstacle (excluding the smooth obstacles). Perceived unpleasantness by (a) roughness and (b) hardness.

**
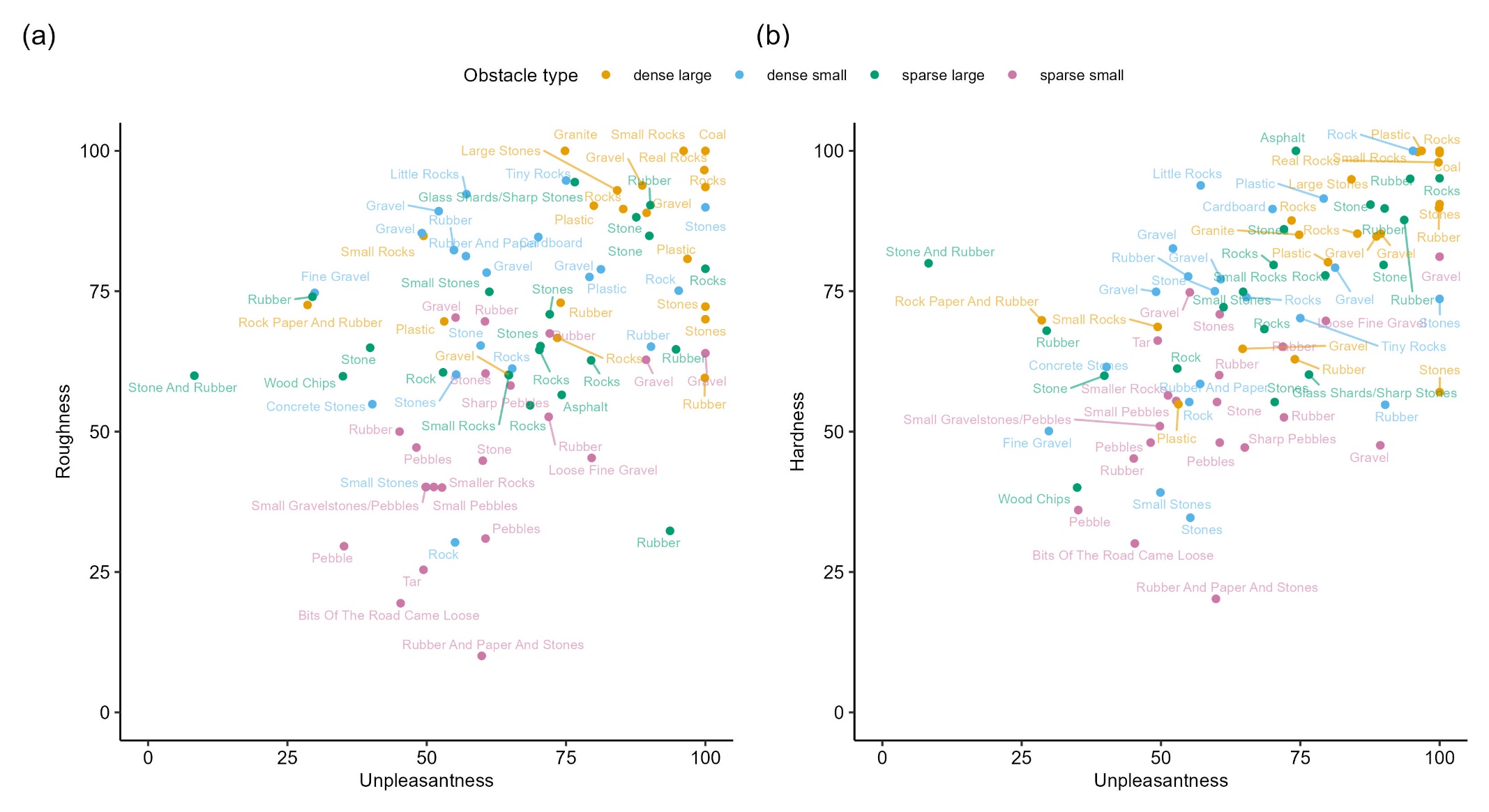
**

**Figure S3**

Crossing step length by (a) trial (linear regression line is fitted to trials) and (b) obstacle type and visual uncertainty. The red shaded region represents the 95% confidence interval and error bars represent ± 1 SEM.

**
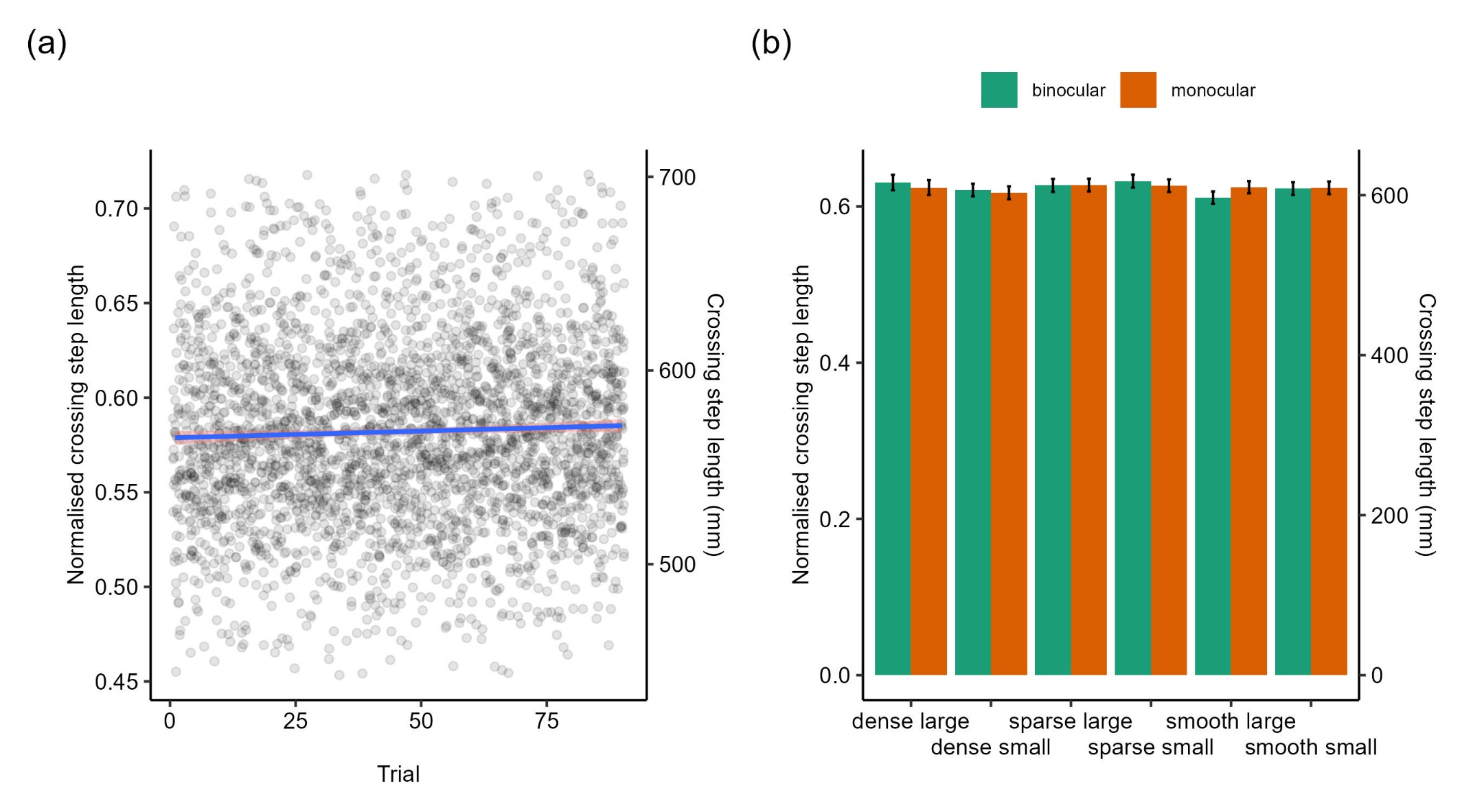
**
